# A method for measuring human body composition using digital images

**DOI:** 10.1371/journal.pone.0206430

**Published:** 2018-11-05

**Authors:** Olivia Affuso, Ligaj Pradhan, Chengcui Zhang, Song Gao, Howard W. Wiener, Barbara Gower, Steven B. Heymsfield, David B. Allison

**Affiliations:** 1 Department of Epidemiology, School of Public Health, University of Alabama at Birmingham, Birmingham, AL, United States; 2 Nutrition Obesity Research Center, University of Alabama at Birmingham, Birmingham, AL, United States; 3 Department of Computer and Information Sciences, College of Arts and Sciences, University of Alabama at Birmingham, Birmingham, AL, United States; 4 Department of Nutrition Science, School of Health Professions, University of Alabama at Birmingham, Birmingham, AL, United States; 5 Pennington Biomedical Research Center, Louisiana State University System, Baton Rouge, LA, United States; 6 Department of Epidemiology and Biostatistics, School of Public Health, Indiana University-Bloomington, Bloomington, IN, United States; University of Tennessee Health Science Center, UNITED STATES

## Abstract

**Background/Objectives:**

Body mass index (BMI) is a proxy for obesity that is commonly used in spite of its limitation in estimating body fatness. Trained observers with repeated exposure to different body types can estimate body fat (BF) of individuals compared to criterion methods with reasonable accuracy. The purpose of this study was to develop and validate a computer algorithm to provide a valid estimate %BF using digital photographs.

**Subjects/Methods:**

Our sample included 97 children and 226 adults (age in years: 11.3±3.3; 38.1±11.6, respectively). Measured height and weight were used (BMI in kg/m^2^: 20.4±4.4; 28.7±6.6 for children and adults, respectively). Dual x-ray absorptiometry (DXA) was the criterion method. Body volume (BV_PHOTO_) and body shape (BS_PHOTO_) were derived from two digital images. Final support vector regression (SVR) models were trained using age, sex, race, BMI for % BF_NOPHOTO_, plus BV_PHOTO_ and BS_PHOTO_ for %BF_PHOTO._ Separate validation models were used to evaluate the learning algorithm in children and adults. The differences in correlations between %BF_DXA_, %BF_NOPHOTO_ and %BF_PHOTO_ were tested using the Fisher’s Z-score transformation.

**Results:**

Mean BF_DXA_ and BF_PHOTO_ were 27.0%±9.2 vs. 26.7%± 7.4 in children and 32.9± 10.4% vs. 32.8%±9.3 in adults. SVR models produced %BF_PHOTO_ values strongly correlated with %BF_DXA_. Our final model produced correlations of *r*_*DP*_ = 0.80 and *r*_*DP*_ = 0.87 in children and adults, respectively for %BF_PHOTO_ vs. %BF_DXA_. The correlation between %BF_NOPHOTO_ and %BF_DXA_ was moderate, yet statistically significant in both children *r*_*DB*_ = 0.70; p <0.0001 and adults *r*_*DB*_ = 0.86; p<0.0001. However, the correlations for *r*_*DP*_ were statistically higher than *r*_*DB*_ (%BF_DXA_ vs. %BF_NOPHOTO_) in both children and adults (children: Z = 5.95, p<0.001; adults: Z = 3.27, p<0.0001).

**Conclusions:**

Our photographic method produced valid estimates of BF in both children and adults. Further research is needed to create norms for subgroups by sex, race/ethnicity, and mobility status.

## Introduction

Assessment of body composition, particularly fat and fat-free mass, is vital to understanding many health-related conditions including cachexia induced by HIV, cancer, and other diseases; multiple sclerosis; wasting in neurological disorders such as Parkinson’s, Alzheimer’s, and muscular dystrophy; sarcopenia; obesity; eating disorders; proper growth in children, and response to exercise[1–7]. Nevertheless, challenges remain in the determination of these aspects of body composition in research[8]. Obesity, characterized by an excess of body fat (BF) and sarcopenia, defined as diminution of primarily skeletal muscle, remain significant public health problems [9, 10]. Both obesity and sarcopenia can be assessed using highly accurate techniques such as dual-energy x-ray absorptiometry (DXA) or magnetic resonance imaging (MRI) but are not widely used in large-scale epidemiologic studies or non-clinical settings due, in part, cost and size of the equipment used for these methods. Furthermore, field methods such as multiple skinfold measurements depend heavily upon repeated training of research staff to obtain accurate and reliable assessments [11]. Therefore, body mass index (BMI; kg/m^2^) is a commonly used alternative, but is limited in that it is an assessment of body weight relative to height and not of body composition per se. It is well-documented that obesity is often misclassified when BMI is used as a proxy for body fatness compared to measurement techniques via imaging. [12, 13]. In children, age- and sex-specific BMI percentiles have been found to underestimate the prevalence of excess adiposity when compared to DXA particularly among whites and Mexican American youth [12]. Similarly in adults, BMI misclassified obesity status differentially by race/ethnicity and age [13]. Therefore, there is significant need for a simple, portable, and relatively inexpensive but accurate measurement of body composition that performs well across age, sex, and racial/ethnic groups.

The use of digital photography may be a viable alternative to BMI for the assessment of human body composition in field research. This method has the potential to overcome limitations associated with BMI particularly the misclassification of obesity among individuals who have relatively high lean mass (i.e. body builders) or low lean mass (i.e. the elderly). Our approach to using digital images to assess human body composition is predicated on evidence from studies done as early as the 1930’s using either visual estimation [11, 14–16]; or photographic assessment of body volume, from which body composition could be determined[17–19]. Previous studies employing visual estimation have found that both trained and untrained observers can provide moderately accurate estimates of percentage BF by visually inspecting an individual directly or from photography with correlations between observations and criterion measures, such as under water weighing (UWW) ranging between r = 0.56 and 0.83. This evidence suggests that visual estimation of body composition can be valid, but may be limited by familiarity of the observer with the study population, subjectivity of the observer, and reproducibility of the study results. Prior to the advent of digital photography, researchers used manual photographic methods to provide valid and reliable estimates of body volume as a means to overcome the substantial expense and participant burden associated with UWW. However, these early attempts still required significant labor to process the photographs and manually calculate body volume. The automation of the visualization process by using computerized digital image analysis could overcome several of the issues associated with visual assessment of body volume and composition. Therefore, the purpose of this study was to develop an easy, portable, quick, and comparatively inexpensive, but valid computerized image analysis method for use in large-scale and/or remote studies to estimate fat and fat free mass.

## Methods

### Study sample

Participants were 323 children and adults aged 6–80 years representing a broad range of shapes and sizes recruited from the metropolitan Birmingham, AL area (2012–2014) via flyers, newspapers, newsletters, an online research referral service, word of mouth, visits to local recreational centers, churches, and community events. Inclusion criteria were: 1) without diseases known to effect body composition; 2) able to stand for three photographs; 3) no contraindication for a DXA body composition scan; 4) not missing more than one finger or toe to reduce error in volume determination; and 5) not pregnant. Participants were instructed to refrain from caffeine and large meals prior to the single study visit. Informed consent was obtained from the adults/parents and children provided their assent for study participation. The study protocol was approved by the Institutional Review Board of The University of Alabama at Birmingham. All participants received $20 for completing the study.

### Measures

All measures were assessed by trained research staff with participants dressed in close fitting but non-compressing LYCRA shorts and tank/sports bra (females only) without shoes. Height (to the nearest 0.1 cm) and weight (to the nearest 0.1 kg) were measured using a physician’s balance beam scale with stadiometer (HealthOMeter—Model 402LB, McCook, IL). BMI (kg/m^2^) was calculated from measured height and weight. Obesity status was categorized in children as BMI≥95 percentile and BMI≥30 in adults, according to standard definition by expert panels [20, 21]. Body composition was measured using DXA (GE Lunar iDXA, Madison, WI) with the pediatric software employed when appropriate (encore 2011 version 13.6). Quality assurance tests were performed daily per the manufacturer instructions. Obesity via DXA was defined as body fat percent ≥ 25% in males, ≥30% girls and ≥35% in women [22, 23]. Three photographs (front, back, and side profiles) were taken with a digital camera (Canon PowerShot—Model SX50; Canon USA Inc., Melville, NY) with participants standing against a photography green screen.

### Body volume and body shape from two-dimensional image processing

Body volume (BV_PHOTO_) and body shape (BS_PHOTO_) were estimated from photographs of each participant using two photographs (back and side profiles). Although we had a third photograph of the front profile, the use of the additional photograph did not change our estimation of body volume or shape. Briefly, the methods used to determine BV and BS are described below. However, our technical report of the detailed methods used herein are described elsewhere [24].

**Fig 1:**
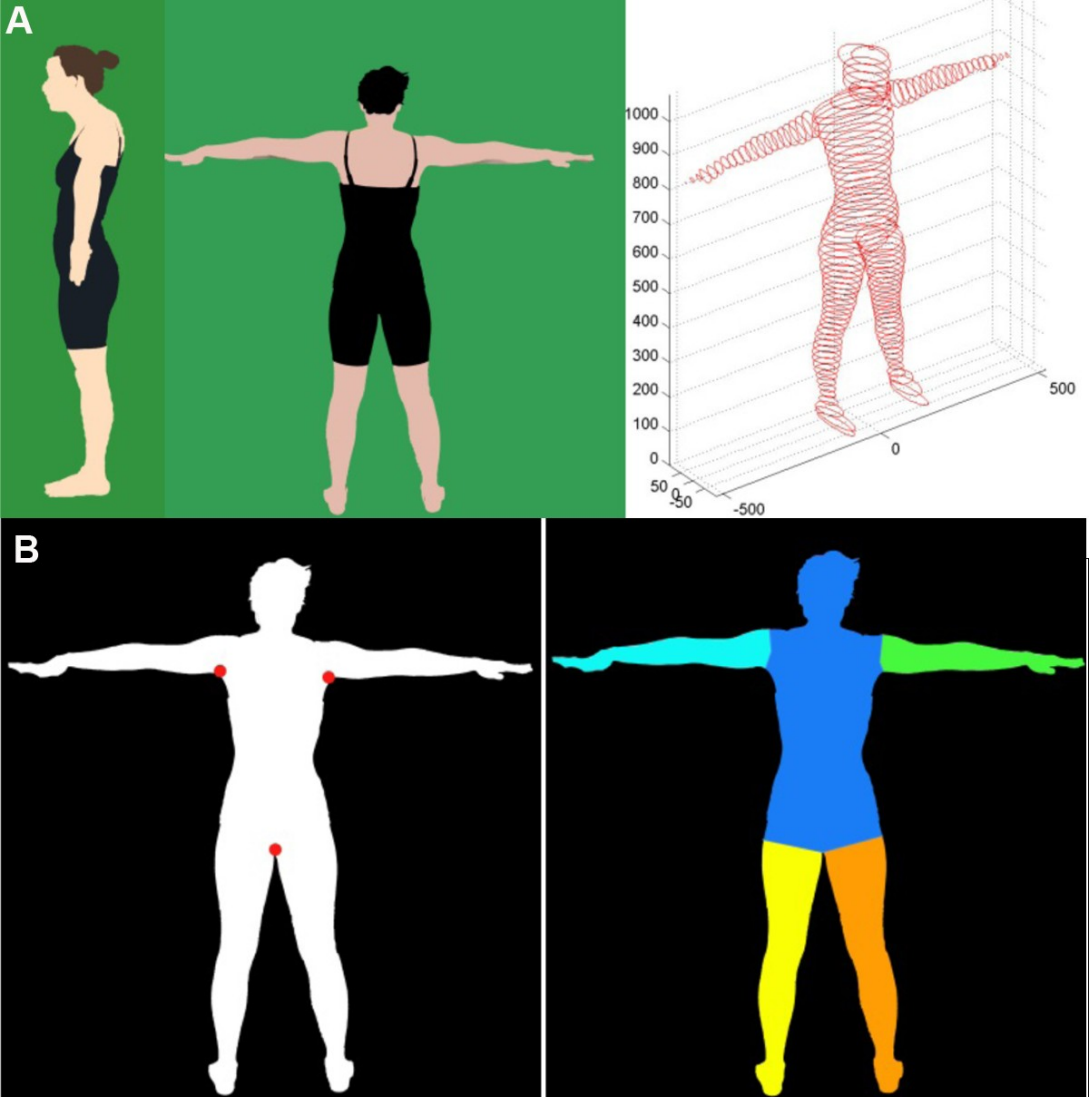
A. 3D-body model from 2D images. B. Key points to separate body segments.

We constructed a 3-dimensional body model based on both the back and side body masks (i.e., outline of the body) extracted from 2D profile images as shown in Fig 1A. The body position for the back profile photograph required participants to stand with their arms and legs separated, while for the side profile photograph the arms were required to be close to the body with the legs together. Also for the side profile, the right leg and foot were covered in a green cloth to isolate the side of the body. The distance of the camera from participants (91 in.) and the camera/lighting settings were standardized to reduce variation in the photographs among participants. A four-step procedure was then used to separate the body components of each participant and was as follows: 1) The body was separated from the green screen by setting a color intensity threshold that facilitated the differentiation between the background and the black clothing and skin tone in the photograph; 2) The body mask from the back and side profiles were rotated until symmetrical in the vertical plane; 3) The height from the body mask was then normalized to represent participants' actual height; 4) Finally, the separation points, called *key points (see* Fig 1B*)*, used to determine the separation line of each body component such as the arms, legs and the trunk, were detected for each participant from the back profile, which allowed better separation due to natural folds of the skin.

The local dimension features (e.g. length and width) of each body component such as arms, legs, and trunk, were used to construct ellipse-like slices along the main orientation of each component. Long and short axes were calculated for each ellipse-like slice depending upon the length and width of each body component. The area of each slice was taken to be the number of pixels within that slice. A 3D body model was then constructed by accumulating ellipse-like slices and the BV_PHOTO_ was derived by summing the area of all slices.

**Fig 2:**
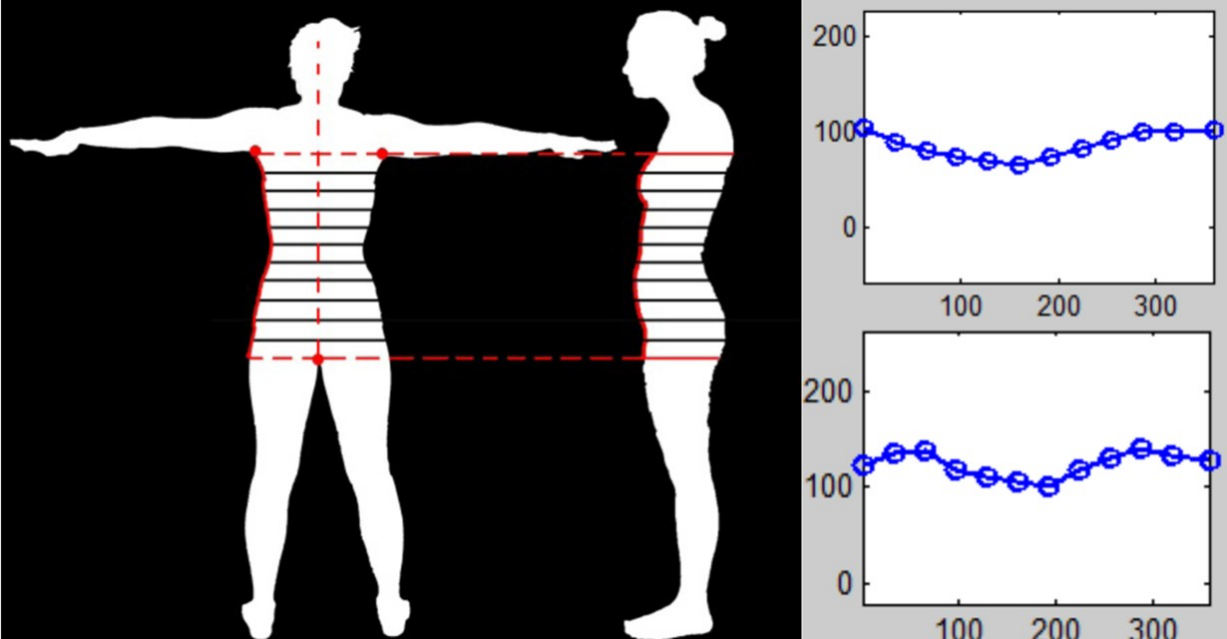
Body shape feature derives from front and side curves.

Body shape features, which delineate fat distribution in the trunk, were captured by extracting the front curve (FC) and the side curve (SC) using the left upper key point and the lower key point as demonstrated in Fig 2. The width of the body was taken at each of 12 equally spaced points along the vertical contour of the front of the body, providing 12 numerical values representing the FC of each person. Likewise, the side curve of the body was measured by 12 additional equally spaced lines representing the extracted side contour. The side contour lines were measured to the central bodyline (CBL, formed by a vertical line drawn from the top of the head through the lower key point) and gave the twelve numerical values representing each person’s SC. Details of the methods used to extract the shape features are published elsewhere [24].

After extracting FC and SC, a cluster evaluation function in MatLab (‘evalclusters’) with K-means clustering and ‘Calinski-Harabasz’ criterion was used to compute optimal number of clusters ‘*k’* for both the FC and SC features from the training dataset [25]. This cluster evaluation step examined cluster sizes from 2 to 10 and computed the optimal cluster size for the FC and SC feature sets. FC and SC were then represented as *k*-element vectors, consisting of 0’s and 1’s. A value of one at the n^th^ element indicates that the body shape is in the n^th^ cluster. The encoded vectors for FC and BC are represented as BS_PHOTO_ in our model and used as categorical features to train the prediction models. After determining the optimal number of clusters and their corresponding cluster-centroids using only the training dataset, FC and SC in the testing dataset were later assigned to the clusters whose centroids were closest in terms of Euclidean distance.

### Building the prediction model

BV_PHOTO_ and BS_PHOTO_ of each participant along with the covariates age, race, sex, BMI were used as input features as shown in the first step of Table 1, to train a support vector regression (SVR) for the estimation of %BF_PHOTO_. The ground truth collection, type of regression model used and the parameter optimization for the regression model are also described in steps two, three and four of Table 1. Separate 3-fold cross validation models were used to evaluate the performance of the learning algorithm for the prediction of body fat in children and adults [26,27]. Both datasets were then shuffled randomly and divided into three equal subsets. The 3-fold cross validations were then conducted by taking two of the subsets as the training dataset and remaining one subset as the testing dataset without any overlap. In addition, we repeated the process three times, choosing separate subsets for testing each time. Hence, we tested each data point in the dataset once after completing one round of the 3-fold cross validation process as described in step 5 of Table 1.

**Table 1:** Procedures used in the support vector regression for the photographic estimation of body fat.

Steps	Detail description
**1. Feature set generation**	Back and side profile images of 323 children and adults ranging from age 6–80 were collected along with their age, sex, race and BMI information. The pictures are used to compute BV_PHOTO_, FC and SC. FC and SC were separately used to cluster the participants into *k* groups which is computed using a cluster evaluation function in MatLab (‘evalclusters’) with K-means clustering and ‘Calinski-Harabasz’ criterion. These clusters represent different body shapes and are represented as BS_PHOTO_ in our study. Hence, our feature set for each participant is: $$\begin{array}{cccccc} age & sex & race & BMI & BV_{PHOTO} & BS_{PHOTO} \end{array}$$
**2. Ground truth**	We also measure the BF% for each participant using a DXA machine (BF_DXA_%). BF_DXA_% will be our ground truth BF% for training and testing the prediction model.
**3. Regression model**	We train Support Vector Regression (SVR) model to predict BF_DXA_ using our feature set. We use the ‘nu-SVR’ type SVR with ‘radial bias’ type kernel function from LIBSVM[31]
**4. Parameter optimization**	‘nu-SVR’ requires several parameters [31] Values of -s and -t are selected to be 4 and 2 respectively. A parameter selection during operation was performed using our training dataset to select the best parameter set for the–g (gamma), -c (cost) and–n (nu). Combinations of g, c and n, within certain ranges were tested training, and the best combination that produced the highest prediction accuracy in the training data set was chosen for our regression model. The values of -g and -c were taken as 2^X^, where X is an integer ranging from -10 to 10. Similarly values of n were taken from 0.1 to 1 at an interval of 0.1.
**5. Training and Testing**	The participants were randomly divided into 3 subsets and 3-fold cross validations were performed by taking two of the subsets as the training dataset and remaining subset as the testing dataset. FC and SC curves from the training dataset were used to compute the *k* clusters. Each test dataset was assigned to the clusters discovered during training by computing the nearest cluster centroid to compute their BS_PHOTO_. This process was repeated 3 times, choosing separate subsets for testing each time.

### Statistical analysis

Descriptive statistics (means, SD, ranges, and/or frequencies) were computed for the participant characteristics stratified by adults and children.

Pearson’s correlations between the DXA criterion measure of body fat and predicted values from a simplified model (BV_PHOTO_ + covariates) and the full (BV_PHOTO_ + BS_PHOTO_ + covariates) model were calculated. The correlations are represented as follows:

Where,

*r*_*DP*_ denotes the correlation between %BF by DXA and % BF by PHOTO,

*r*_*DB*_ denotes the correlation between %BF by DXA and %BF by BMI plus covariates.

We used the method of Meng et al.[28] to determine whether our method (i.e. %BF_PHOTO_) was significantly better correlated to the criterion method of %BF_DXA_ than was %BF_NOPHOTO_[29]. We also created Bland-Altman plots of the difference in estimation of body fat by each method to examine the error in our estimation across the range of %BF_DXA_ and %BF_PHOTO_ measurements [30].

SVR models were constructed using LIBSVM[31] in MatLab Version R2014b (Mathworks, Inc., Natick, MA). Correlation and the test for statistical difference were computed using SAS Version 9.3 (SAS, Inc., Cary, NC). Alpha level of significance was set at p<0.05, 2-tailed).

## Results

Participant characteristics stratified by children (6–18 years) and adults (≥19 years) are presented in Table 2. Our sample included 97 children and 226 adults (mean ± SD of age in years: 11.3±3.3; 38.1±11.6, respectively). Mean BMI and %BF_DXA_ were 20.4 kg/m^2^±4.4; 28.7 kg/m^2^±6.6 and 27.1%±9.2; 32.7%±10.4 for children and adults, respectively). Among the children, 47.4% were female, 60.8% were African American (AA), while among the adults, 46.5% were female and 46.5% were AA. Also, 14.4% of children and 33.6% of adults were classified as obese using BMI (age- and sex-specific BMI≥95^th^ percentile for children and ≥30 kg/m^2^ for adults), while 40.2% of children and 66.4% of adults were over the threshold for body fatness via DXA (≥30% for girls and ≥25% for boys; ≥35% for women and ≥25% for men.

**Table 2:** Sample characteristics.

	Adults n = 226	Children n = 97
Age (mean, SD), years	38.1 (11.1)	11.3 (3.3)
Percentage Female (n, %)	105 (46.5)	46 (47.4)
Percentage African American (n, %)	105 (46.5)	59 (60.8)
Height (mean, SD), cm	169.2 (8.4)	148.5 (15.4)
Weight (mean, SD), kg	82.2 (20.1)	46.3 (16.2)
BMI (mean, SD), kg/m^2^	28.7 (6.6)	20.4 (4.4)
BMI Percentile (mean, SD)	-	64.8 (27.6)
DXA Body Fat (mean, SD), %	32.9 (10.4)	27.0 (9.2)
Photographic Volume (MP)	25.4 (6.3)	14.1 (4.9)

MP = megapixel

**Fig 3:**
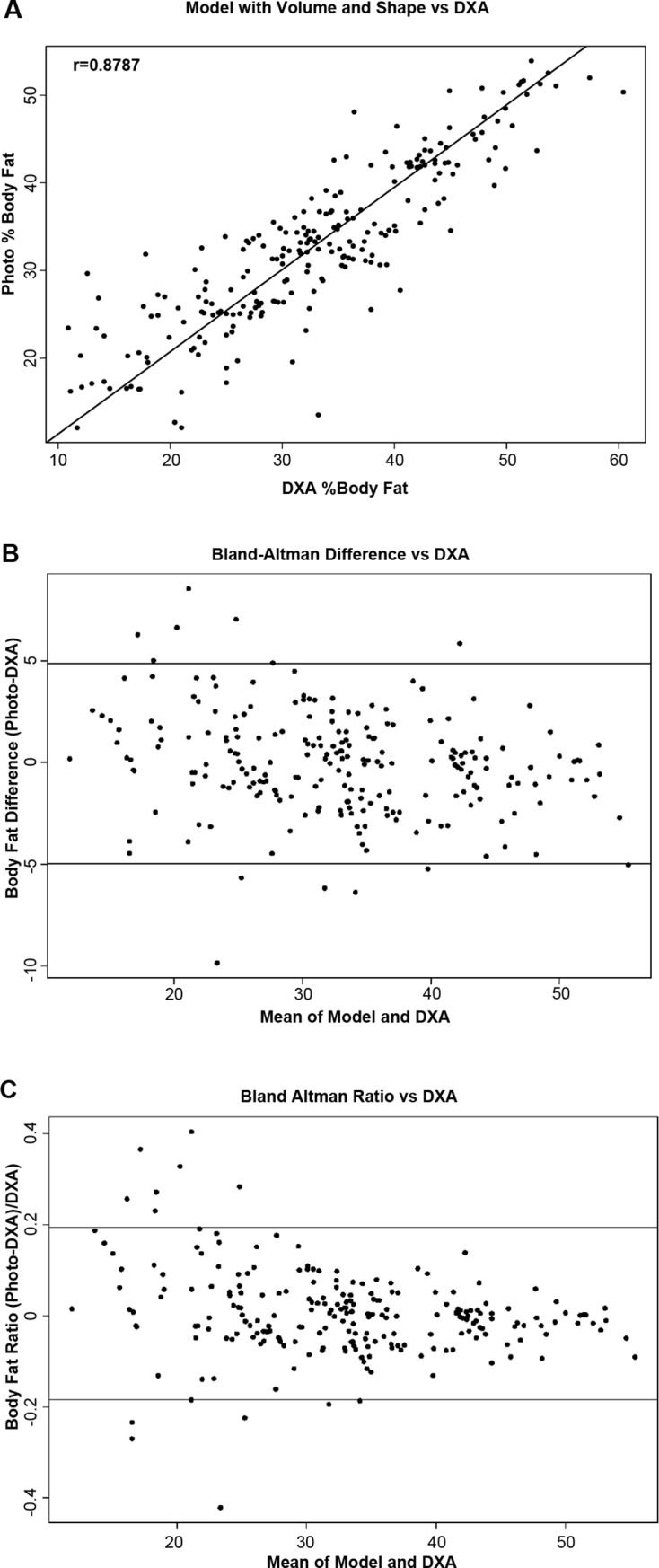
Correlations between predicted body fat from the photographs and dual-energy x-ray absorptiometry (DXA). (A) and Bland-Altman plots of the absolute (B) and relative (C) differences between the two methods (horizontal lines represent the 95% confidence intervals) among adults.

**Fig 4:**
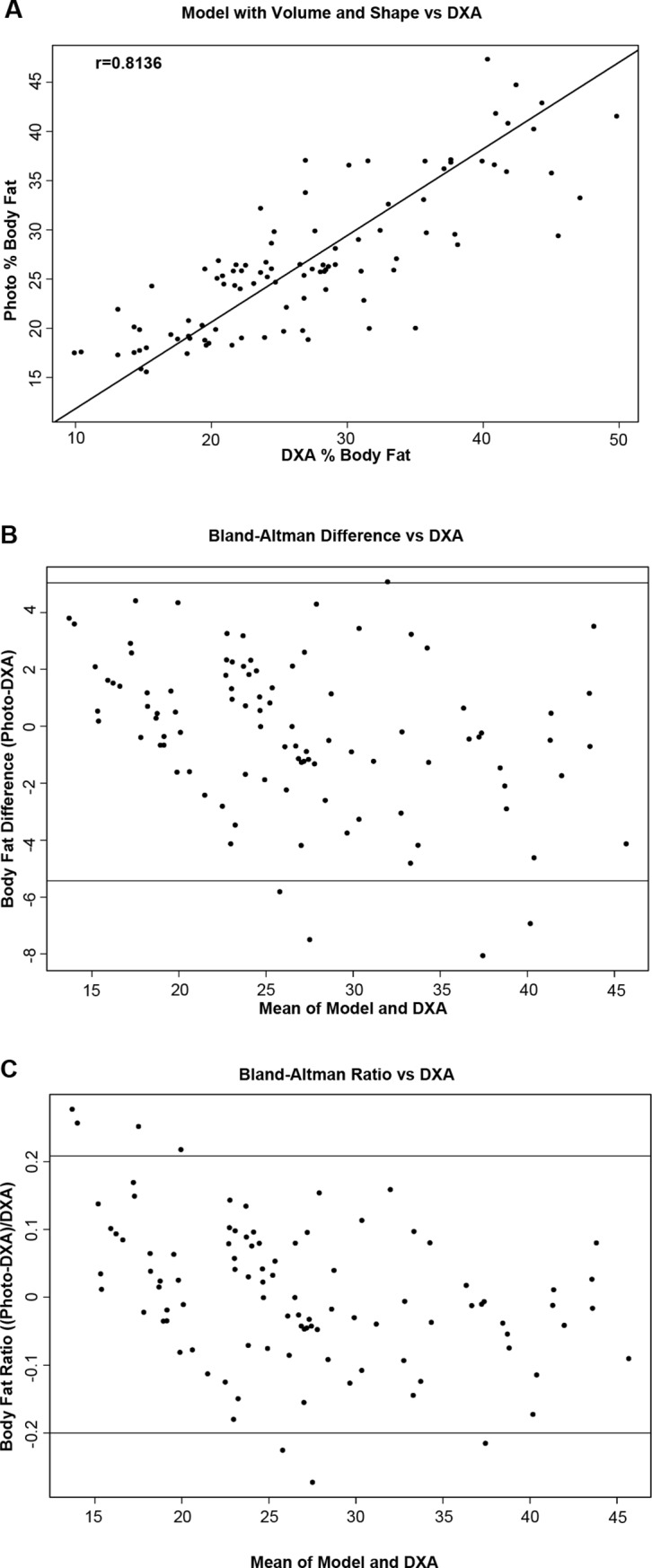
Correlations between predicted body fat from the photographs and dual-energy x-ray absorptiometry (DXA). (A) and Bland-Altman plots of the absolute (B) and relative (C) differences between the two methods (horizontal lines represent the 95% confidence intervals) among children.

Correlations between %BF_DXA_ and the %BF_PHOTO_ from our learning algorithms are presented for adults and children in Figs 3 and 4, respectively. Both SVM models produced %BF_PHOTO_ values that were strongly correlated with the %BF_DXA_. %BF_PHOTO_ from Model 1 which included demographic variables plus BMI and BV_PHOTO_ produced correlations of *r*_*DP*_ = 0.72 and *r*_*DP*_ = 0.86 in children and adults respectively. However, %BF_PHOTO_ from Model 2 which included variables from Model 1 plus BS_PHOTO_ produced *r*_*DP*_ = 0.81 in children and *r*_*DP*_ = 0.88 in adults.

The correlations among %BF_DXA_, %BF_NOPHOTO_, and %BF_PHOTO_ are reported in Table 3. The correlation between %BF_NOPHOTO_ and %BF_DXA_ was moderate, yet statistically significant in both children (*r*_*DB*_ = 0.70;p < 0.0001) and adults (*r*_*DB*_ = 0.86;p < 0.0001). *r*_*DP*_ was significantly larger than *r*_*DB*_ in both children and adults (children: *Z* = 5.95; p < 0.0001; adults: *Z* = 3.27; p < 0.001). Bland-Altman plots revealed greater differences between our %BF_PHOTO_ and the %BF_DXA_ in the tails of the distributions in both adults and children (Fig 3A and 3B). In adults, the mean absolute and relative differences between the two methods were -0.06 (95% CI: -4.97, 4.85) and 0.005 (95% CI: -0.18, 0.19), respectively. In children, the mean and absolute and relative differences were -0.19 (95% CI: -5.43, 5.04) and 0.004 (95% CI: -0.20, 0.21), respectively.

**Table 3:** Correlations and concordance between DXA and estimated body fat.

	Adults n = 226		Children n = 97	
	DXA % Body Fat	Lin’s Concordance Coefficient	DXA % Body Fat	Lin’s Concordance Coefficient
**Non-Photographic % Body Fat**	0.86^a^	0.85	0.70^HYPERLINK^ ^a^	0.66
**Photographic % Body Fat (Volume)**	0.86	0.85	0.72	0.67
**Photographic % Body Fat (Volume and Shape)**	0.88^b^	0.87	0.81^b^	0.79

**DXA =** dual energy x-ray absorptiometry

^a,b^Differences in Correlation Coefficients between non-photographic model and photographic model containing volume and shape: Adults—Z = 3.27, P<0.001; Children—Z = 5.95, P<0.0001. Lin’s Concordance Coefficients are comparisons of the model estimates of % body fat and DXA % body fat.

## Discussion

Innovations in both digital photography and image analysis have allowed the automation of visual body composition assessment. Our study results showed strong correlations between predicted body fat percentage derived from 2D digital photographs and body fatness from DXA in a sample of children and adults. The performance of our image analysis algorithm provided statistically better correlations to DXA measurements than containing BMI and demographic information. The average absolute error between our method and DXA was small (~4.1%) with BV and BS contributing significantly to the overall prediction of percent body fat.

Our estimates of body volume from the photographs were highly correlated with body volume from air displacement plethysmography suggesting that our photographic method is valid for use in the prediction of body composition (r = 0.98)[32]. To further enhance our estimation of percent body fat, we incorporated visual elements of body shape that were also shown to correspond to levels of adiposity from DXA (i.e. thin body shapes corresponded to low body fatness). The clustering of body shape and percent body fat suggests that body shape provides important visual information for improving the accuracy of the image analysis algorithm and may have implications on the assessment of health outcomes beyond overall adiposity. The findings followed a similar pattern among children and adults though the correlation between our method and DXA was higher in adults.

Moreover, our results are consistent with other studies that use multiple cameras (e.g. 8–16 cameras) to estimate BF in humans [33, 34]. The simplicity of our method overcomes several issues associated with other field measures of body composition such as portability, ease of data collection, and expense. Relative to a DXA machine (~$30,000 USD), the setup cost for our photographic method is ~$300, which includes the cost for a scale/stadiometer, Chromakey green background, tank top & shorts, and digital camera. Our preliminary software program was has been developed in Matlab and will require the end-user to enter demographic information along with the selection of a front and side photograph previously saved on the computer. Furthermore, our method requires little human input during the image capture and processing procedures, thereby reducing biases inherent in other field methods such as visual estimation and skinfold measurement, which require training and re-training[11, 14–16, 35]. Lastly, no specialized equipment is required beyond a simple digital camera and a green backdrop makes our portable method more convenient than other methods.

### Implications

Computerized image analysis of digital photographs can be used as a valid method for estimating body fatness in humans. Simple digital photographs processed with our algorithm could be used in place of BMI alone in both clinical practice and public health research. This is of particular interest in research as evidence suggests that associations between obesity assessed by BMI and outcomes such as mortality may be different (e.g. Linear vs U-shaped) when a more robust method of body composition is used. Our novel method also addresses the need for a portable, yet valid, method for measuring body composition in large epidemiological studies as well as investigations conducted in remote locations.

### Strengths & limitations

The strengths of this study include the use of digital photographs to capture unbiased information about the body for use in the estimation of volume and shape, DXA as the criterion measure of body composition, and a diverse sample of black and white youth and adult males and females. However, several limitations of this work should be also noted, including the small sample within each stratum of race, sex and age as well as the inclusion of only able-bodied individuals who could stand for the photographs. Although attempts were made to include a broad range of body sizes at this stage in the development of this method, there were limited numbers of participants enrolled in the tails of the distribution for this analysis. Therefore, the results are not necessarily generalizable to very lean or very obese individuals, all race-ethnic groups or persons unable to stand for the photographs. Future studies should examine the performance of our photographic method in a larger sample to allow stratification by age, sex, and race/ethnicity.

## Conclusion

This research shows that a computer algorithm can be developed to provide a valid estimate of body fatness from 2D photographs taken with a regular digital camera.
